# Teenage recommendations to improve physical activity for their age group: a qualitative study

**DOI:** 10.1186/s12889-018-5274-3

**Published:** 2018-03-20

**Authors:** Michaela James, Charlotte Todd, Samantha Scott, Gareth Stratton, Sarah McCoubrey, Danielle Christian, Julian Halcox, Suzanne Audrey, Elizabeth Ellins, Samantha Anderson, Isabel Copp, Sinead Brophy

**Affiliations:** 1 College of Medicine, Data Science Building, Swansea University, Singleton Park, Swansea, SA2 8PP UK; 2 College of Engineering, Bay Campus Swansea University, Fabian Way, Crymlyn Burrows, Skewen, Swansea, SA1 8EN UK; 3 City and Council of Swansea, Room 153, Guildhall, Swansea, SA1 4PE, Swansea, UK; 4 Department of Sport and Physical Activity, Edge Hill University, St Helens Road, Ormskirk, Lancs, L39 4QP, Wolverhampton, UK; 5 Swansea University, Singleton Park, Swansea, SA2 8PP UK; 6 Office Room 4.02, Canynge Hall, 39 Whatley Road, Bristol, BS8 2PS UK; 7 Birchgrove Comphrehensive School, Birchgrove Rd, Birchgrove, Swansea SA7 9NB, Swansea, UK; 8 Cefn Hengoed Community School, 60 Caldicot Rd, Bon-y-maen, Swansea SA1 7HW, Swansea, UK

**Keywords:** Physical activity, Recommendations, Teenagers, Barriers

## Abstract

**Background:**

It is recommended that young people should engage in 60 min of moderate-to-vigorous activity (MVPA) a day for health benefits, but few teenagers actually meet this recommendation. Policy-makers play a vital role in designing physical activity initiatives, but they generally do this with little or no input from the intervention recipients. This study explores the recommendations made by teenagers to improve activity provision, uptake and sustainability of physical activity engagement for both themselves and their peers.

**Methods:**

Thirteen focus groups were carried out in seven secondary schools in South Wales, United Kingdom. Participants (*n* = 78) were recruited from a larger mixed-method randomised control trial, which involved the implementation of a voucher scheme to promote physical activity in teenagers (aged 13–14). Thematic analysis was undertaken to identify key issues from the perspective of the teenage participants.

**Results:**

Six key recommendations were identified following analysis of the focus groups: i) Lower/remove the cost of activities without sacrificing the quality, ii) Make physical activity opportunities more locally accessible, iii) Improve the standards of existing facilities, iv) Make activities more specific to teenagers v) Give teenagers a choice of activities/increase variety of activity and vi) Provide activities that teenage girls enjoy (e.g., fun, sociable and not competitive sport). Throughout the focus groups, the increased opportunity to participate in unstructured activity was a key recommendation echoed by both boys and girls in all themes.

**Conclusion:**

There is a disconnect between what is available and what teenagers want to do. Policy-makers and those involved in physical activity delivery (e.g., schools, local council and local activity providers) should include young people in designing interventions and facilities to ensure they are meeting the needs of this age group and providing the right opportunities for teenagers to be active. That is unstructured, local, low cost, fun, sociable opportunities and the right facilities to be active.

**Electronic supplementary material:**

The online version of this article (10.1186/s12889-018-5274-3) contains supplementary material, which is available to authorized users.

## Background

It is recommended that young people should engage in 60 min of moderate-to-vigorous activity (MVPA) a day for health benefits [1], yet many fail to meet this recommendation [2–4]. It is estimated that 80% of teenagers worldwide are not sufficiently active [5]. Physical activity has been found to positively impact on both physical and psychological health [3, 6–10]. The main barriers to being active for teenagers are reported to be cost, accessibility and lack of local facilities [11–17]. Many physical activity interventions have chosen to focus on these to underpin their approaches to activity promotion [3, 11, 18–20]. Policy-makers play a vital role in designing physical activity initiatives but they often have little or no input and feedback from key intervention recipients. This creates a ‘policy gap’ between professional understandings of young people’s health needs and what teenager’s actually want from interventions [21].

Involving target populations in policy-making processes is said to increase legitimacy, justifiability and feasibility over policies designed through more traditional, top-down methods [22]. Initiatives designed in this way are noted to be more responsive to user’s needs and improve the quality of their aims [23, 24]. When discussing activity with teenagers, research has shown that there is a difference between current activity provision and what young people actually want and recommend [11, 14, 15]. Therefore, involving teenagers in the design and implementation of physical activity initiatives may be key in influencing the activity uptake of among this age group [25, 26].

The Active Children through Incentive Vouchers – Evaluation (ACTIVE) Project [27] centres upon user involvement, through the provision of physical activity vouchers to all pupils in year nine in four intervention schools. The project gives pupils the choice over access to existing provisions or support in designing their own [11]. The baseline data collection for the project involved focus groups with 13–14 year old pupils in seven schools (four intervention and three control schools). The focus group interviews were conducted to include teenagers in the design of the ACTIVE intervention and give them the opportunity to make their own recommendations to tackle inactivity in young people. The aim of this was to understand the current barriers to physical activity faced by teenagers and understand potential ways in which teenagers feel these barriers could be overcome. The interviews were conducted prior to the ACTIVE intervention, to establish what was missing for teenagers in their local area and what could be done to combat inactivity.

Previous research has focused on adult involvement in the policy making process in clinical settings [21–23, 28, 29]. The recommendations made by teenagers could increase the success of physical activity policies and initiatives and help improve the short and long-term health of young people. This study explores the recommendations made by teenagers to improve activity provision, uptake and sustainability of physical activity engagement for both themselves and their peers. Through doing so, further understanding can be gained of the current barriers, facilitators and motivation [30–32] to being active faced by 13–14 year olds attending secondary schools in deprived areas of Wales.

## Methods

Thirteen focus groups were carried out in seven secondary schools in South Wales, United Kingdom. Participants (*n* = 78) were recruited by purposive sampling to ensure a mix of genders from a larger mixed-method randomised control trial, which involved the implementation of a voucher scheme to promote physical activity in teenagers (aged 13–14). Schools were approached to take part due to their: i) location in one of Wales’ most deprived areas and ii) location in a Communities First catchment area [33]. Schools were randomly assigned into either the intervention or control arm of the trial.

Focus groups were selected as the preferred methodology due to their distinguishing feature of group interaction, which can encourage in-depth discussion. The groups consisted of 6–8 pupils selected at random. Boys and girls were mainly in separate groups to establish any gender differences in discussions and recommendations in terms of motivation to be active [30] or differences in activity preferences [34, 35]. This was also because of the trend for girls of this age to drop out of physical activity at a higher rate than boys [34] and therefore, separate focus groups could provide into why this is the case. As a result, two focus groups were conducted in each school, except for one school where, due to time constraints, boys and girls were combined to make one focus group.

Focus groups were carried out at the schools during the school day to ensure pupils remained in a comfortable, familiar setting and lasted 30–60 min (the average length was 38.42 min). A lead moderator, previously experienced in leading focus groups, facilitated the focus groups to allow detailed discussion of the teenager’s recommendations and gain a better understanding of their needs; improving the quality of ACTIVE’s aims [36]. An assistant moderator was also present at each focus group and was responsible for taking notes and audio recording. The assistant moderator was also responsible for reporting back to participants on their main discussions from the focus groups to ensure correct interpretation and understanding and gain clarity over any points discussed, a method of respondent validation [37] and increasing the trustworthiness of the findings. The pupils involved in the focus groups had previously met the moderator/assistant moderator during data collection at the school. To ensure consistency across all focus groups, a semi-structured topic-guide was used, which reflected the study’s aims [38]. This can be found as Additional file 1. This was to provide triggers for discussion rather than a prescriptive structure. Once transcribed in verbatim, NVivo 10 was used to manage, code and analyse the data and two researchers validated the themes derived from the data via triangulation [2]. This was done after the coding process. To protect participants identities, names were removed during transcription. Participants were encouraged to review themes in order to validate findings.

Thematic analysis (TA) was undertaken in order to identify, analyse and report patterns within the discussion with the teenage participants without the rigidity and inflexibility of other qualitative analysis methods [39, 40]. This analysis approach was used due to the ability of TA to examine the different perspectives of the participants, as well as its usefulness to summarise key points of a large data set [40] helping to produce a clear conclusion, particularly in this instance where many viewpoints needed to be considered. This is due to TA forcing the researcher to take a well-structured approach to handling data [40].

Braun and Clarke’s Phases of Thematic Analysis (2006) [41] was used to underpin the coding process. Once familiarised with the transcripts of the focus groups, initial codes were generated, defined and named accordingly with data collated into the relevant theme. This process was ongoing throughout the analysis phase to refine the specifics of each theme and the overall story the analysis told [41]. The analysis was carried out by two researchers independently who compared coding/themes in order to guarantee no new codes/themes have emerged and there are instances of the same theme to ensure data saturation [42]. This also helped achieve inter-rater reliability. Lincoln and Guba’s (1985) criteria for trustworthiness was used to ensure trustworthiness by using an audit trail, method and analyst triangulation [2]. Consolidated criteria for reporting qualitative research (COREQ) guidelines were used to inform the analysis and presentation of this study [43].

### Ethical approval

All participants provided written assent and parental consent for participation in focus groups. Participation was voluntary and participants were informed of their right to withdraw from any aspects of the study at any time. The College of Human and Health Science Ethics Committee at the College of Medicine, Swansea University granted the ACTIVE Project ethical approval on 12/05/2016 (Reference: 090516).

### Findings

Themes emerged following Braun and Clarke’s Phases of Thematic Analysis (2006) [41]. Initially, 17 codes were used across the entire data set with these collated into six key recommendations based on similarities in phrases/words used by participants. This was the point in which data saturation occurred and no new codes emerged when researcher’s compared analysis.

The six recommendations to improve physical activity for teenagers were: i) Lower/remove the cost of activities without sacrificing the quality, ii) Make physical activity opportunities more locally accessible, iii) Improve the standards of existing facilities, iv) Make activities more specific to teenagers, v) Give teenagers a choice of activities/increase variety of activity and vi) Provide activities that teenage girls enjoy (key quotes from the focus groups are in Table 1).

**Table 1:** Six Key Recommendations to Improve Teenage Activity

Teenage recommendations	
Lower/remove of the cost of activities without sacrificing the quality	
“And probably if like the leisure centres dropped their prices, you know, maybe people will think, oh that’s cheaper, okay I’ll go back.” (Girl, Focus Group 6)	
“What they could do is like get like something in a park… if they say it’s like a free thing they would all just like come in and do it, instead of… if they say it’s like £3 to come in they’d be like oh okay, bye.” (Boy, Focus Group 13)	
“...if it was like free and all that you’d see loads of other older kids going to try it out because they know it’s free and it’s something to do with their friends, and they don’t have to spend their own money.” (Boy, Focus Group 13)	
“…they’ve got like one indoor pitch which costs a lot to play in, or they’ve just got outdoor pitches which are, like, really cheap to play but it’s, like, really cold. They don’t put, like, any lights on.” (Boy, Focus Group 9)	
“…I feel bad when I have to go up to my parents and ask them for money, because their face is just like, right, and then you can see them as they pass the money over and they don’t like it.” (Girl, Focus Group 4)	
Improving the locality of physical activity opportunities	
“Well I would say bring more facilities, bring more stuff so then like more football pitches, basketball pitches, like more stuff so they’re going to want to go outside.” (Boy, Focus Group 5)	
“Just like a little gym, in the park or something, ‘cos I would go then ‘cos it’s like really close.” (Girl, Focus Group 6)	
“So there’s the travel, but if it was, like, in your community, then it wouldn’t be so bad.” (Girl, Focus Group 10)	
“They could spend more money and invest in putting buildings in that area where they could put, for example, badminton, tennis, football, rugby” (Girl, Focus Group 8)	
Improving the standards of existing facilities	
“Like we said, like, fix the parks and stuff like that.” (Girl, Focus Group 10)	
“I think they could like, well not even like every year, like every other year they could go round to each park and renew all the apparatus.” (Boy, Focus Group 5)	
“And in the gym there’s umm a few of the machines are broke, you could pay to get them fixed or like help get new ones and stuff like that.” (Boy, Focus Group 7)	
“They need to make the environment better.” (Girl, Focus Group 10)	
“But why don’t they invest in building more things down there for our age because I, you walk through there and you mostly see glass bottles on the floor, on benches and…” (Boy, Focus Group 9)	
“Yeah, council investing in, like, one-way systems and everything and they’re wasting money on build, on making these one-way systems and everything when they could be looking at our age and start investing in buildings that we can go to and enjoy ourselves after school.” (Boy, Focus Group 9)	
Make activties more specific to teenagers	
“And they always do adult things, like they never really aim at anything for teenagers, like people our age.” (Girl, Focus Group 6)	
“Yeah, the government is complaining saying that we’re getting like, there’s like less people being fit but there’s not really more facilities and stuff for like teenagers.” (Girl, Focus Group 6)	
“No, and like I just think they should put more activities out for younger people, like…” (Girl, Focus Group 6)	
“For our age group and under 16’s, not so much adults…” (Boy, Focus Group 7)	
Give teenagers a choice and variety of activities	
“There’s like clubs on, it’s the exact same every single time you go.” (Girl, Focus Group 2)	
“…they should give you a sheet at the beginning of the year and then choose which ones you want to do and then they go with the majority…” (Girl, Focus Group 12)	
“Rather than doing the same thing, like football, hockey, you know…” (Girl, Focus Group 4)	
“Yeah, but they could take us out of our PSE when we have it and then maybe at dinner times?”(Boy, Focus Group 1)	
“Yeah I think it’s as much quantity as it is quality.” (Boy, Focus Group 13)	
Provide activities that teenage girls can enjoy	
“If I don’t like it, I won’t do it.”(Girl, Focus Group 10)	
“You could hold like a football game but then for the people who like football and then for the people who like cheerleading they could let them cheerlead, or people who like dancing and things you could just hold a massive event of sports and have people performing.” (Girl, Focus Group 12)	
“Make sure, like get their friends to do it as well, so then their friends can encourage them, like oh I’m going to go there, oh come with me, be like oh okay. Ask them.” (Girl, Focus Group 12)	
“Yeah, just ask them if they want to go swimming, like say it’s a normal thing, ‘cos nobody would think of swimming as like an active thing isn’t it, just for fun” (Girl, Focus Group 6)	

### Lower/remove of the cost of activities without sacrificing the quality

Teenagers identified reducing the cost of being active as a key recommendation. They suggested that there could be a reduction made to the existing price of activities in order to increase accessibility and sustainability. A boy stated that "...if it was like free and all that you’d see loads of other older kids going to try it out because they know it’s free and it’s something to do with their friends, and they don’t have to spend their own money" (Boy, Focus Group 13). Another girl reiterated this point by saying "…and probably if like the leisure centres dropped their prices, you know, maybe people will think, oh that’s cheaper, okay I’ll go back" (Girl, Focus Group 6).

One girl explained how she would have to ask for money from her parents in order to access activities and this would make her feel bad, as she knew her parents were reluctant to pay. This would deter her from being active. She said "…I feel bad when I have to go up to my parents and ask them for money, because their face is just like, right, and then you can see them as they pass the money over and they don’t like it" (Girl, Focus Group 4). Free activities were recommended as an alternative approach; however, teenagers were aware that there is sometimes a trade-off in quality in exchange for lower priced activities. One boy explained that "…they’ve got like one indoor pitch which costs a lot to play in, or they’ve just got outdoor pitches which are, like, really cheap to play but it’s, like, really cold. They don’t put, like, any lights on" (Boy, Focus Group 9).

If the facilities are without heat or are dirty or unsafe due to low investment this will not encourage activity hence, purely lowering cost, without maintaining the quality of provision would not have the desired effect in enhancing teenage activity levels. One way to tackle this is to offer informal activities in a good quality venue, as these incur less cost to run and attend [16]. This would include offering self-directed gym sessions, unstructured football sessions where teenagers can attend and play without coaching, or provision of any venue where a qualified coach is not required to teach technical movements or referee. This focus on quality of facilities is also re-iterated in the theme pertaining to improving the standards of existing facilities.

### Improve local access to physical activity opportunities

Throughout the focus groups, it was evident activities should be made more local to where teenagers lived. Similar to the theme of cost, improving access to activity has repeatedly been expressed as a barrier [3, 11, 14, 15]. Teenagers advocated for closer proximity of facilities, commenting that they would be more inclined to access activities that are closer to their homes. One boy said, "I would say bring more facilities, bring more stuff so then like more football pitches, basketball pitches, like more stuff so they’re going to want to go outside" (Boy, Focus Group 5). A girl noted that the proximity of “…a little gym, in the park or something” would help her be active “…‘cos it’s like really close” (Girl, Focus Group 6). This was particularly relevant to outdoor spaces like pitches and parks. Teenagers suggested that they need to travel to be able to play outdoors, and this would incur an additional cost.

Removing the need for travel to venues, would go some way to making physical activity more accessible to these teenagers. Both boys and girls provided examples of specific equipment and/or facilities to increase physical activity such as more local *“football pitches, basketball hoops”* and *“little gyms in the park”.* It was apparent in the focus groups that both teenage boys and girls were happy to organise their own activity if provided with the facilities, as they did not mention the need for formal coaching in these activities. This suggests teenagers would like the increased opportunity and space to participate in unstructured, non-competitive forms of their favourite sports.

### Improving the standards of existing facilities

When teenagers discussed their local facilities, they noted their current standards are in need of improvement. This was due to facilities, such as parks, falling into states of neglect and equipment being broken. This conversation focussed on local parks but also extended to discuss gym equipment and the aesthetic features of facilities (e.g., lighting). There were small differences in the way in which boys and girls felt this maintenance could be done. Boys recommended buying new equipment to replace the old, while girls discussed improving what is already there for example, one girl said, "Like we said, like, fix the parks and stuff like that" (Girl, Focus Group 10). One boy stated, "I think they could like, well not even like every year, like every other year they could go round to each park and renew all the apparatus" (Boy, Focus Group 5). Another boy noted the ways in which the council has been investing in other provisions that he did not feel was important, he said, “…yeah, council investing in, like, one-way systems and everything and they’re wasting money on build, on making these one-way systems and everything when they could be looking at our age and start investing in buildings that we can go to and enjoy ourselves after school” (Boy, Focus Group 9). It was apparent among focus groups with both genders that local facilities are lacking. The council’s control of local provision was frustrating for teenagers because they felt more should be invested to maintain the environment and improve local facilities. It was evident that what is already in the community is not appealing to teenagers due to lack of general maintenance.

By improving and updating local activity provision, teenagers say they are more likely to access them. Their recommendations propose that the local council need to be more proactive in their monitoring and upkeep of facilities. There was a mutual feeling among boys and girls that the local council is avoiding investing in teenagers and have chosen to invest in other developments, such as road maintenance, which teenagers do not value. This point also draws out the need for activities and facilities invested in to be useable and appealing to teenagers and relates strongly to the next theme of ensuring activities provided are specific to teenagers.

### Make activities more specific to teenagers

Both girls and boys commented on making activities more age-relevant. Girls in one focus group discussed the ways in which activity provision does not target their age group and wanted more *“encouragement”* or to clearly be included and invited*.* There is very little that specifically invites teenagers or promotes and provides where they feel it is for them. One girl stated "…they always do adult things, like they never really aim at anything for teenagers, like people our age" (Girl, Focus Group 6). Another boy echoed this by saying he wanted to see more activity provision for “…our age group and under 16’s, not so much adults…” (Boy, Focus Group 7).

The provisions suggested by these teenagers included whole gyms designed for their age group and the ability to be able to attend existing classes for example, currently, there are age restrictions on classes like Zumba and Yoga. The teenagers believed the local council has neglected their age group, one girl said "…yeah, the government is complaining saying that we’re getting like, there’s like less people being fit but there’s not really more facilities and stuff for like teenagers" (Girl, Focus Group 6). Boys also acknowledged the lack of provision for their age group, noting that that most provision is aimed at adults.

### Give teenagers a choice and variety of activities

Teenagers in most of the focus groups recommended that they have a choice over which activities are available for their age group. In terms of local community provision, they wanted *“quantity as well as quality”,* allowing them to access a broad variety of activities. The focus groups made it evident that local activity provision is lacking in variety and teenagers do not get a choice as to what activities they would like to do. One girl said that “there’s like clubs on, it’s the exact same every single time you go” (Girl, Focus Group 2). While another girl requested that activity provision should be varied “rather than doing the same thing, like football, hockey, you know…” (Girl, Focus Group 4). Like the “improving locality of physical activity” theme, the activities suggested to provide variety were unstructured. For example, one participant suggested they would like more choice to be able to play non-conventional sports like dodgeball in an unstructured format, where they could organise teams and rules themselves.

This lack of choice and variety was evident in the school setting too. The girls discussed this lack of choice in detail, suggesting they were more disengaged with school sport than the boys were. Girls discussed how inflexible Physical Education (PE) lessons were to providing variety and suggested giving each pupil a sheet at the beginning of the year with which they could suggest/pick activities they would like to do. They noted that schools provide traditional, structured forms of sport, whereas they would prefer more unstructured activities. One girl suggested that “…they should give you a sheet at the beginning of the year and then choose which ones you want to do and then they go with the majority…” (Girl, Focus Group 12).

The boys discussed being able to have the ability to choose when they could be active, for example, being able to come out of other lessons to do so. For the boys, it was more of a case of being able to choose to do more activity rather than being discontent with the activity already on offer.

### Provide activities that teenage girls can enjoy

It was apparent when discussing types of activity, that teenage girls are more likely to be active if they can access activities they enjoy. It was evident that if they do not like what is on offer, they will not participate in it and would prefer to be inactive. One girl said “if I don’t like it, I won’t do it” (Girl, Focus Group 10). The idea of being able to enjoy activity was prominent amongst the girls in the focus groups and a greater emphasis was placed on the enjoyment aspect of activity among girls throughout discussions. It was important for girls that the purpose of the activity was not to ‘be active’ per se, rather they preferred the emphasis to be on the opportunity for them to have fun. The examples of activities that fit this criteria were the local waterpark (with slides and wave machines) and a trampoline park because *“it’s fun,”* yet still gets teens active. One focus group also suggested the idea of a girl’s only gym in which girls could be the only ones allowed to access it which would make the experience more enjoyable as being red and sweaty in front of boys was described as a barrier.

Inclusivity was a big part of this theme as girls suggested that everyone has a role to play in activities. These different roles included unstructured forms of activity such as cheerleading for school sports teams, which could be led and organised by teenagers. One example of how this could be done was suggested by a girl who said, “You could hold like a football game but then for the people who like football and then for the people who like cheerleading they could let them cheerlead, or people who like dancing and things you could just hold a massive event of sports and have people performing” (Girl, Focus Group 12). Inclusive activities would also mean peers could be active together allowing more time to spend with friends and facilitate social networks, which was appealing for teenagers [13].

## Discussion

This study aimed to explore the recommendations made by teenagers to improve activity provision, uptake and sustainability of physical activity engagement for both themselves and their peers. The focus groups identified six key themes that would be important to consider in order to improve the success of physical activity policies and initiatives for young people. The study suggests that cost, accessibility and lack of local facilities are perceived by teenagers to be barriers to physical activity as confirmed in other research publications [11–17]. Previous studies have found short-term improvement to physical activity levels when purely addressing the barriers to being active, [19], particularly in the school setting [3, 11, 20]. However, the repeated acknowledgment of these barriers in this study suggests that despite a number of initiatives implemented to tackle these obstacles, the issue has not been adequately addressed long-term.

Throughout the focus groups, the increased opportunity to participate in unstructured activity was a key recommendation echoed by both boys and girls in all themes. This is noteworthy as previous interventions have offered structured activity such as coached dance lessons to combat inactivity, however these have only seen short-term improvements to physical activity and do not show evidence of sustainability [3]. There was no mention of coaches, teams or leagues but there was a universal agreement that activity should allow teenagers the opportunity to enjoy and choose what they would like to do with their friends.

It was clear from this study that current activity provision is not meeting the wants and needs of young people. Teenagers feel frustrated, not encouraged and disengaged with local physical activity provision. This lack of choice means teenagers are bored and disengaged with their local provision as there is difference between what is offered and what teenagers would like to do [24]. For example, teenagers suggested they wanted access to nice facilities for little to no cost and no oversight therefore it could be an idea to increase the accessibility to leisure centres or improve the facilities in local parks so that teenagers can go to the gym or play football with their friends in pleasant environments. This could be as simple as the local council organising an evening where teenagers can use their gyms for free or at a reduced rate. Teenagers would feel valued and allow them to have more choice in what they can do in their local areas.

Involving teenagers in the design and implementation of physical activity initiatives in this way is imperative in empowering teenagers to positively influence their activity levels. Therefore, acknowledging and gaining a better understanding of teenagers’ own recommendations and needs would increase the legitimacy and feasibility of activity interventions as agreed with by previous literature involving the public in designing public health initiatives [21, 22]. The recommendations highlight the importance of relevance, choice and motivation for teenagers. Motivation, in particular, is an important correlate and determinant of physical activity [31]. The importance of acknowledging the different types of motivation to be active cannot be understated as this would help policy-makers understand why teenagers choose to be active and tailor initiatives to suit motivations. Self-Determination Theory (SDT) [44] has emerged as a popular framework for examining motivation and physical activity [31] as it differentiates between controlled motivation (e.g., regulated by external control or guilt) and autonomous motivation (e.g., regulated by enjoyment and personal values) [30]. The recommendations made suggest teenagers are motivated autonomously due to their focus on enjoyment and personal values of spending money, for example. This is positively related to sustained health behaviours [31]. Therefore, by addressing autonomous motivation, policy-makers are more likely to promote physical activity behaviours that would be valued and sustained by young people. Consequently, addressing accessibility, specificity of activity, choice and enjoyment is paramount to improving teenage activity levels.

During the focus group discussions, there were a few subtle gender differences to emerge. For example, girls placed more emphasis on the enjoyment aspect of activity and the need to be active with friends. Girls also seemed to be more disengaged with school PE than boys, something that has been acknowledged by previous initiatives [10, 17]. More girls are believed to have negative experiences in PE that lower interest and involvement in physical activity in their leisure time [45]. These findings suggest that a focus on reviewing physical activity provision for girls in secondary schools may go some way towards addressing girls’ physical activity levels (e.g., allowing them a choice of activities to choose from). This is particularly important as declines in physical activity levels amongst girls are greater than in boys [34]. Gender differences have been acknowledged in physical activity interventions, however they have either been unsuccessful or positive outcomes have been short-lived [3, 34, 46]. This may be due to not considering what motivates teenage girls. For example, implementing a school-based intervention with dance as the activity will not be successful if girls do not enjoy dance. Hence, while certain aspects of physical activity interventions may need to be tailored to specific genders (e.g., greater emphasis on selling the enjoyment and socialisation aspect of the intervention for girls), the overall core components need not differ. Focusing on reducing cost, improving locality and standards of physical activity facilities, lowering age limits on activities and providing choice and variety is likely to enhance participation for both boys and girls in this age group.

### Limitations

Whilst the use of focus groups enabled a more in-depth exploration of teenager’s barriers to physical activity, the focus groups were conducted with a limited age range and only those children consenting to take part in the study were able to be involved in the focus groups. These children could potentially be the more active and involved children, perhaps not capturing the views of those less engaged with activity and health. Furthermore, the focus groups were conducted with a limited age-range of teenagers (aged 13–14 years old), this means the recommendations made by teenagers aged 15 years old and upwards have not been included and may differ. One focus group was conducted with both boys and girls together, which may also have affected the recommendations made from this particular focus group.

## Conclusion

Teenagers believe current physical activity provisions should be low cost, should be local, are in need of improvement, should be specific to their age, need more choice/variety and need to include activities that they enjoy. Based on the recommendations made by teenagers in this paper, physical activity interventions could be influenced and designed more effectively. For example, the six recommendations could be used as a guide for future activity regarding activity levels in young people. In particular, interventions need to consider the motivations of teenagers in reference to Self-Determination Theory [44] as a guiding principle in their development. They should consider whether the group they are targeting are motivated in a controlled or autonomous manner in order to be more effective. Therefore, a consultation phase, could go a long way to improving physical activity in certain groups. As previously mentioned, involving target populations in policy-making processes is said to increase legitimacy, justifiability and feasibility over policies made through more traditional, top-down methods [22].

Throughout the focus groups and spanning across all themes, the increased opportunity to participate in unstructured activity was echoed by both boys and girls in all themes. There was no mention of coaches, instructors, teams or leagues. However, there was a universal agreement that activity should allow teenagers the opportunity to enjoy and choose what they would like to do with their friends. Key examples of this were accessing the local trampoline and water park. In these environments, teenagers can organise their own activity and define their own teams and rules. Therefore, if allocated the correct facilities, resources and opportunities, teenagers believe they would be more active.

Policy-makers and those involved in physical activity delivery (e.g., schools, local council and local activity providers) should include young people in designing interventions and facilities to ensure they are meeting the needs of this age group and providing the right opportunities for teenagers to be active. By acknowledging the recommendations made in this paper, physical activity initiatives can improve uptake, sustainability and overall success of future projects. The ACTIVE Project [27] will use these recommendations to underpin its delivery of a physical activity intervention for young people in Wales focusing upon user involvement in its design and implementation.

## Additional file

Additional file 1: ACTIVE Focus Group Topic Guide PUBH-D-17-02284R2. ACTIVE Focus Group Questions The topic guides for the ACTIVE focus groups for both intervention and control schools. (DOCX 14 kb)

## Abbreviations

ACTIVE: Active Children Through Individual Vouchers Evaluation
COREQ: Consolidated criteria for reporting qualitative research
MVPA: Moderate to vigorous physical activity
PE: Physical education
SDT: Self-determination theory
TA: Thematic analysis
