# Supplementary material for: Teenage recommendations to improve physical activity for their age group: a qualitative study
Source: BMC Public Health. 2018 Mar 20;18:372. doi: 10.1186/s12889-018-5274-3 (PMC5859389; doi:10.1186/s12889-018-5274-3)
Supplement: Supplementary file 1 — ACTIVE Focus Group Topic Guide PUBH-D-17-02284R2. ACTIVE Focus Group Questions The topic guides for the ACTIVE focus groups for both intervention and control schools. (DOCX 14 kb) [file 12889_2018_5274_MOESM1_ESM.docx]

**ACTIVE Focus Group Questions**

**Intervention Group**

*Activity 1 (5 – 10 mins):* What is physical activity? What does it mean to you?

*(Flipchart/post-its – ask pupils to discuss/write down what the term means to them)*

How active should people your age be? (60 minutes recommended per day)

*Question 1:*  How active do you think people in your year are? Do you think you are as active as you can be?

*Activity 2 (5 – 10 mins):* What do you see as the current barriers to physical activity? How do you feel about your current levels?

*(Give post-it notes and ask pupils to list 5-6 barriers to activity. Then rank these barriers in order of most common barriers and discuss the reasoning for this order)*

*Question 2:*  Why do you think people your age like/don’t like being active? Is there much to do in your area? If so, how accessible are these activities to your age group?

*Question 3:* Vouchers are currently accepted by *(refer to list of participants).* What other activities or providers would you like to see included before we start?

*Question 4:*  Having heard the way the scheme is set up; do you think there will be any problems? What do you think we could do about these problems? Do you think we should do things differently?

*Question 5:* What is the best way of letting everyone in the school know about this scheme? When are the best times for the support worker to be available in the school?

*Question 6:* Do you have anything else to add about the project?

**ACTIVE Focus Group Questions**

**Control Group**

*Activity 1 (5 – 10 mins):* What is physical activity? What does it mean to you?

*(Flipchart/post-its – ask pupils to discuss/write down what the term means to them)*

How active should people your age be? (60 minutes recommended per day)

*Question 1:*  How active do you think people in your year are? Do you think you are as active as you can be?

*Activity 2 (5 – 10 mins):* What do you see as the current barriers to physical activity? How do you feel about your current levels?

*(Give post-it notes and ask pupils to list 5-6 barriers to activity. Then rank these barriers in order of most common barriers and discuss the reasoning for this order)*

*Question 2:*  Why do you think people your age like/don’t like being active?

*Question 3:* Is there much to do in your area? If so, how accessible are these activities to your age group?

*Question 4:*  What do you think the best way to get people active is? What needs to happen in your local area?

*Question 5:*  Do you have anything else to add?
